# Honest signaling in domestic piglets (*Sus scrofa domesticus*): vocal allometry and the information content of grunt calls

**DOI:** 10.1242/jeb.138255

**Published:** 2016-06-15

**Authors:** Maxime Garcia, Marianne Wondrak, Ludwig Huber, W. Tecumseh Fitch

**Affiliations:** 1 Department of Cognitive Biology, University of Vienna, Althanstrasse 14, Vienna 1090, Austria; 2 Comparative Cognition, Messerli Research Institute, University of Veterinary Medicine Vienna, Medical University of Vienna, University of Vienna, Veterinaerplatz 1, Vienna 1210, Austria; 3 Haidlhof Research Station, 2540 Bad Vöslau, Austria

**Keywords:** Domestic pig, Acoustic allometry, Longitudinal study, Formants, Size information, Vocal communication

## Abstract

The information conveyed in acoustic signals is a central topic in mammal vocal communication research. Body size is one form of information that can be encoded in calls. Acoustic allometry aims to identify the specific acoustic correlates of body size within the vocalizations of a given species, and formants are often a useful acoustic cue in this context. We conducted a longitudinal investigation of acoustic allometry in domestic piglets (*Sus scrofa domesticus*), asking whether formants of grunt vocalizations provide information concerning the caller's body size over time. On four occasions, we recorded grunts from 20 kunekune piglets, measured their vocal tract length by means of radiographs (X-rays) and weighed them. Controlling for effects of age and sex, we found that body weight strongly predicts vocal tract length, which in turn determines formant frequencies. We conclude that grunt formant frequencies could allow domestic pigs to assess a signaler's body size as it grows. Further research using playback experiments is needed to determine the perceptual role of formants in domestic pig communication.

## Abstract (precis)

**Summary**: Longitudinal investigation of acoustic allometry in domestic piglets indicates that grunt formant frequencies allow conspecifics to assess a signaler's body size.

## INTRODUCTION

Identifying the type of information conveyed by animal acoustic signals is a central research focus in the field of bioacoustics (Bradbury and Vehrencamp, 1998). Studies conducted on different model species have shown that diverse information concerning a caller’s traits may be encoded within the acoustic signal it produces. Vocalizations may thus allow receivers to evaluate many relevant attributes of the caller, including body size (Charlton et al., 2009a; Pitcher et al., 2012; Reby and McComb, 2003; Vannoni and McElligott, 2008), sex (Charlton et al., 2009a; Vignal and Kelley, 2007), age (Charlton et al., 2009a; Reby and McComb, 2003), individual identity (Charlton et al., 2011a, 2009b; Reby et al., 1998; Robisson et al., 1993), group membership (Boughman, 1997; Randall et al., 2005), geographical origin (Catchpole and Armanda, 1993), motivational state (Kreutzer et al., 1999), physical condition (Wyman et al., 2008), hormone levels (Charlton et al., 2011c; Koren and Geffen, 2009) and emotional state (Briefer, 2012).

Among these topics, the particular study of ‘acoustic allometry’ has recently emerged, focusing on identifying the vocal correlates of a caller's body size (Fitch, 2000c; Reby and McComb, 2003; Rendall et al., 2005). Because body size has a fundamental influence on animal ecology (Peters, 1983), physiology (Taylor et al., 1982) and social behavior (Clutton-Brock et al., 1979; Ryan, 1980), accurate acoustic cues to body size should be biologically relevant, and not only perceived but also interpreted and utilized by receivers.

In birds and mammals, early work suggested that fundamental frequency (hereafter *F*_0_), a key component in many acoustic signals, might be negatively correlated with body size, and thus that an impression of bigger size would be conveyed by a lower *F*_0_ (Morton, 1977). This suggestion seems plausible based on the anatomical–physical description of sound production: *F*_0_ corresponds to the rate of vibration of the vocal folds, and longer, thicker vocal folds vibrate at a lower rate (Titze, 1994). If vocal fold length correlated with body size, it would thus be possible to predict a caller's body size based on *F*_0_. However, this acoustic feature has been shown to poorly reflect the caller's body size in various mammalian species (Lass and Brown, 1978; Masataka, 1994; Pfefferle et al., 2007; Rendall et al., 2005), probably due to the absence of strict anatomical constraints on the size of the larynx, which can thus grow with relative independence from overall body size (Fitch and Hauser, 1995).

Unlike the laryngeal structures, the dimensions of the supralaryngeal vocal tract (hereafter simply ‘vocal tract’) are often more closely linked to those of the rest of the body (Fitch and Hauser, 1995). The shape and length of the volume of air within the vocal tract enhance certain resonant frequencies, called formants, and both formants and formant spacing (the mean frequency spacing between consecutive formants) are inversely correlated with vocal tract length (VTL). Formant-related features have been shown to be a good indicator of body size in multiple species (Charlton et al., 2011b, 2009a; Fitch, 1997; Harris et al., 2006; Reby and McComb, 2003). Even when particular adaptations have led to an exaggerated VTL (Fitch and Reby, 2001), formant characteristics can still correlate with VTL and remain a robust and honest indicator of body size within the species because all individuals are subject to the same physical limits imposed by body size (Reby and McComb, 2003).

Research investigating acoustic allometry typically involves cross-sectional studies, sampling a specific group of subjects at a fixed point in time (Evans et al., 2006; Fitch, 1997; Hauser, 1993; Rendall et al., 2005; Riede and Fitch, 1999). For example, a cross-sectional study conducted on humans (Fitch and Giedd, 1999) looked at vocal allometry at different life stages (childhood, puberty and adulthood) and showed that key differences between VTL in males and females arose at puberty, caused by a male-specific laryngeal descent. Although a descended larynx is not typically found in mammals and was previously thought to be uniquely human (Lieberman, 1984), it has recently been reported in non-human primates (chimpanzee; Nishimura et al., 2003), artiodactyls (red and fallow deer; Fitch and Reby, 2001), Mongolian gazelle (Frey and Gebler, 2003), goitered gazelle (Frey et al., 2011), marsupials (koala; Charlton et al., 2011b) and some carnivores (lion, tiger, jaguar, leopard and snow leopard; Hast, 1989; Weissengruber et al., 2002). Additionally, cineradiographic observations on several mammalian species have shown that the larynx is more mobile than previously thought (Fitch, 2000b). Allometric relationships between body size and formants may be affected by larynx descent, whether it occurs at a given point in life or while an animal is vocalizing. However, the importance of acoustic allometry in relation to vocal ontogeny and laryngeal descent/position remains little explored.

List of symbols and abbreviations

AIC: Akaike information criterion
B: basion
BW: body weight
E: base of the epiglottis
Fn: PCA component on *F*_1_ and *F*_2_
*F*_0_: fundamental frequency
*F*_1_: first formant
*F*_2_: second formant
I: incision
P: prosthion
PCA: principal component analysis
S: intersection between nasal tract and apical segment of the piglet snout
VF: vocal folds
VTL: vocal tract length (PCA component on skull length, nasal tract length and oral tract length)
Δ*F*: formant spacing

In this context, domestic pigs (*Sus scrofa domesticus*) represent an excellent model species to examine acoustic allometry, because they are extremely vocal and social, and produce abundant low-frequency and relatively broadband grunts (Kiley, 1972) (ideal for formant salience; Fitch and Hauser, 1995). Within a pig group, size and dominance status are normally strongly correlated (Jensen, 2002), so if cues to body size are present in the formants of pig grunts, they should be highly relevant for receivers. In the present study, we investigated acoustic allometry longitudinally in domestic piglets from the kunekune breed as they grew, making multiple measurements of the same individuals at different life stages. To our knowledge, this is the first longitudinal acoustic allometry study. We captured radiographs (X-rays) of awake piglets and collected body weight data and acoustic recordings of grunts as they aged in order to quantify the anatomical–acoustical correlations relevant to allometric relationships, focusing on formants. As cineradiography data previously collected on a domestic piglet showed only a slight variation of the larynx position while emitting grunts (as opposed to piglet screams, which typically involve laryngeal retraction; Fitch, 2000b), we expected a close relationship between VTL and overall body size, and we predicted that formant characteristics in grunts would provide reliable information regarding the caller's body size in this species. We discuss our findings in relation to the domestic pig's complex communication system, and consider the potential selective advantages of cue extraction in acoustic signals for the receiver.

## MATERIALS AND METHODS

### Study site and animals

The subjects were 20 kunekune piglets (*Sus scrofa domesticus* Erxleben 1777) from three different litters [litter B: *N*=7 (3 females, 4 males); litter R: *N*=6 (4 females, 2 males); litter Z: *N*=7 (2 females, 5 males)] at the Haidlhof Research Station in Bad Vöslau, Austria. Subjects were between 8 and 131 days old during the course of the study. They were housed in semi-natural free-ranging conditions in an 8 ha pasture and a forested patch where five A-shaped huts, a muddy wallow and the water supply were located. The animals had continuous free access to pasture and forest where they spent the nights or found shelter. The pigs lived together in a stable natural social structure, consisting of sounders of three sows and their offspring of two consecutive years, 41 pigs altogether (22 females, 19 males). The subjects of this study were the youngest three litters. Animals were fully habituated to humans (a high number of interactions on a daily basis) and had *ad libitum* water and grass to graze. Additionally, they were fed daily with a diverse mixture of fruits, vegetables, bread and grain.

### Data collection

Piglets were born on 20 June 2015 (litters B and Z) and 22 June 2015 (litter R). Data collection occurred on four different occasions (hereafter ‘series’), namely when piglets were on average 9, 43, 72 and 130 days old (weaning occurred at about 80 days). Body weight (BW) curves from the previous generation were used to evaluate variation in growth rate and select appropriate dates to capture the measurement series. The first three series covered the pre-weaning period, when the piglets' BW increase was not linear, whereas the fourth series occurred after weaning when the piglets' BW increase was stable over time. All piglets were weighed on each of the four series with a My Weigh WR-12K Washdown Scale (reading accuracy, ±20 g) when they were less than 10 kg (series 1–2), and later with a Soehnle 7858 Veterinary scale (reading accuracy, ±100 g accuracy) as soon as some of the piglets weighed more than 10 kg (series 3–4).

### Acoustic recordings

Vocalizations were recorded 10 cm to 1.5 m away from the subjects with a Sennheiser ME-66 directional microphone (frequency response, 40–20,000 Hz ±2.5 dB; Sennheiser Electronic GmbH & Co. KG, Wedemark, Germany) powered by an LR6 battery, and connected to a Zoom H4N digital recorder (48 kHz sampling frequency and 16-bit quantization; Zoom Corporation, Tokyo, Japan). These recordings were stored as uncompressed WAV files. For shock and wind-noise reduction, the microphone was mounted on a Rycote Modular Windshield (Stroud, UK) WS 7 Kit for Shotgun Microphones. Recordings were carried out in a sheltered hut regularly used by the animals, which provided ideal recording conditions (minimal wind and background noise). All 20 individuals were led individually to the hut and had their calls recorded on each of the four series. Recordings were obtained either on the same day or 1 day prior to or following radiograph collection; time constraints prevented collection of both types of data in a single day.

The typical vocalizations recorded from piglets were grunts, as these common low-frequency calls highlight formants better than squeals. For the first series, grunt vocalizations were elicited by preventing the piglets from exiting the hut (blocking the way with the experimenter's hand) or by holding them briefly (which at first elicited squeals, followed by grunts upon their return to the floor). Once piglets were old enough to feed on solid food (from the second series onwards), food was presented as a stimulus to which piglets would produce grunts. This food reward was used in addition to the daily food supply and the *ad libitum* grazing possibility provided by the pasture (no food restriction was imposed, and only the piglets' preference for particular foods was utilized to obtain recordings of grunts, which were then rewarded by several food items during a given recording series).

### Radiographs

Animals were placed in a restrainer, made of Plexiglas for the first series and a hand-made piece of fabric for the following three series (to avoid discomfort as piglets grew older and heavier). Mid-sagittal radiographs of the head and neck region were made with a mobile digital X-ray system, using a full bridge inverter (Physia Gamma light AD 100/120) with different acquisition settings depending on animal size and tissue thickness (series 1: 64 kV, 2.8 mA; series 2: 68 kV, 3.2 mA; series 3: 68 kV, 3.6 mA; series 4: 74 kV, 3.2 mA). Scaling was automatically recorded on the digital radiograph imaging plates used for image capture. All 20 individuals were radiographed on the first and last series. Because of time and logistic constraints, half of the individuals (*N*=10) were radiographed in series 2, and the other half in series 3 (piglet selection was based on BW distribution, chosen to span a measurement range representative of the entire group).

### Data analysis

#### Acoustic measures

**Fig. 1.**
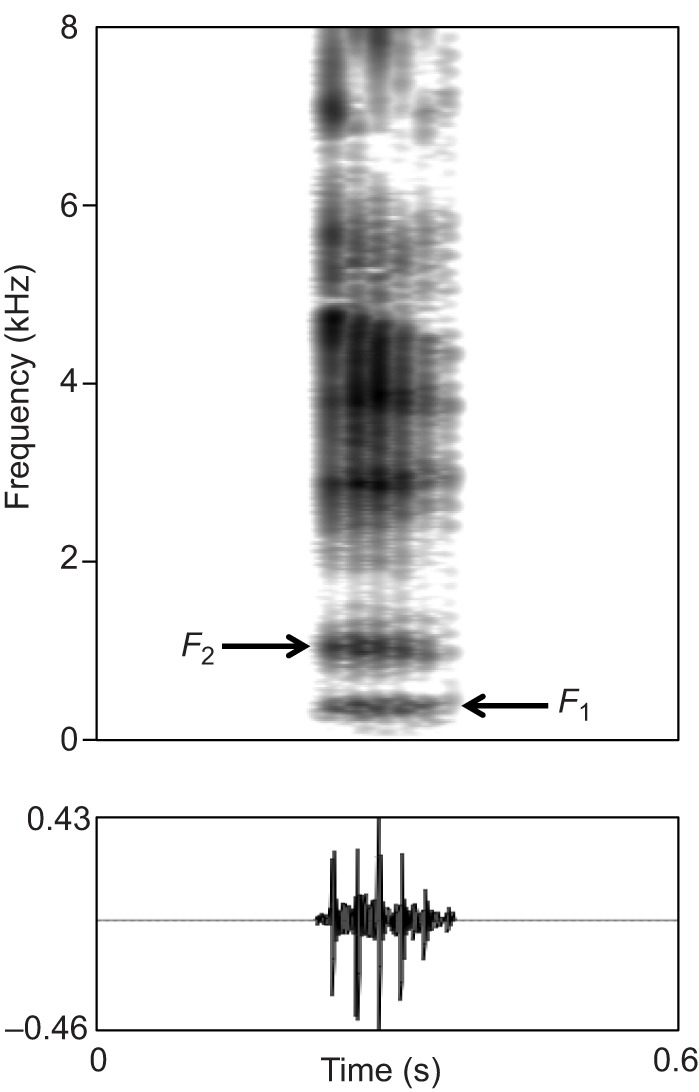
**Spectrogram of a grunt showing its first two formants.** Individual: Baldur; series 4; *F*_1_=409 Hz; *F*_2_=1052 Hz. In most cases (unlike this grunt), formants higher than *F*_2_ could not be clearly distinguished. Visualization settings: view range, 0–8 kHz; window length, 0.04 s; time steps, 700, frequency steps, 250, Gaussian window; dynamic range=40 dB.

All acoustical analyses were made in Praat (P. Boersma and D. Weenink 2014: http://www.praat.org/). Based on both visual inspection of spectrograms and listening, only high-quality grunts (i.e. those deemed to have a high enough signal-to-noise ratio and visible formants) were annotated with ‘Individual’ and ‘Series' using the ‘Annotate: To TextGrid’ function. Care was taken to identify true grunts clearly, as opposed to ‘grunt–squeals' which have quite different acoustic characteristics (Garcia et al., 2016). Annotated grunts were extracted and average formant values were retrieved from each call via a custom-written Praat script (M.G.) that used linear predictive coding (LPC) via the ‘LPC: To Formants (Burg)’ function and allowed editing of the formant contour via the ‘Down to FormantGrid’ function. Formant editing allowed us to remove sections to which Praat automatically attributed a formant value to background noise although the section actually lacked vocalization. Our analysis parameters differed across series and were based on visual inspection of the spectrograms [window of analysis: 0.025 s; time step: 0.00625 s (one-quarter of window length); maximum number of formants: series 1=3, series 2=4, series 3=4, series 4=2, maximum formant frequency, series 1=4500 Hz, series 2=4500 Hz, series 3=4000 Hz, series 4=1500 Hz]. These input settings were adjusted so that formants 1 (*F*_1_) and 2 (*F*_2_) could be distinctly identified and extracted for each series (Fig. 1).

Higher formants were not extracted as they could not reliably be clearly identified in most cases (at least 89%), for two reasons. First, higher formants did not appear to be consistently as well defined as *F*_1_ and *F*_2_. Second, tracking accuracy for higher formants appeared to be affected by slight vocal tract adjustments (both by potentially changing formant contours and spacing and/or by introducing ‘nasal zeros’ or ‘antiformants’, such as seen in humans (Kurowski and Blumstein, 1987)). Ultimately, we retained five grunts per series and per individual, from which we extracted *F*_1_ and *F*_2_ and calculated the average *F*_1_, *F*_2_ (Table S1A–D) and formant spacing (defined here as the average spacing between *F*_1_ and *F*_2_; hereafter, Δ*F*) for each individual within each series. Whenever more than five calls per individual and per series were available, we performed a second, stricter quality assessment and if this was not sufficient to narrow the sample down to five, we made a random selection of five calls among the remaining highest quality files. Overall, only three individuals in series 1 did not have sufficient good quality recordings to reach the criterion of five calls; these cases were therefore excluded from the analysis.

#### Radiographic measurements

**Fig. 2.**
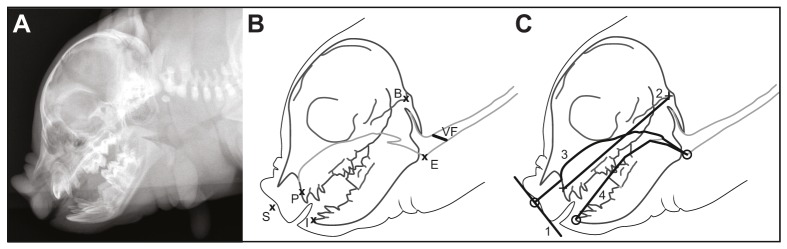
**Illustration of the measurements obtained from landmarks placed on radiographic images.** (A) Radiograph of a domestic piglet (individual: Bolero; first series). (B) Landmarks used to measure vocal tract length (VTL) from radiographs. P, prosthion, the most anterior portion of the maxilla between the incisor roots; I, incision, located at the incisal level of the lower central incisors; B, basion, the midline anterior margin of the foramen magnum; E, base of the epiglottis; S, projection from the nasal airway onto the snout apical line (see C); VF, position of the vocal folds as estimated from anatomical data. ‘I’ was chosen over the lower jaw equivalent of the prosthion because the latter point could not always be identified. ‘E’ was chosen over the location of the vocal folds themselves, as they were rarely clearly observed on radiographs because of the low absorbance difference between soft and calcified tissues in these young animals (although their expected position is indicated in B, based on anatomical images of sectioned piglet heads (W.T.F., unpublished data). (C) Illustration of the measurement taken from radiographs: 1, apical line; 2, proxy of skull length (straight-line distance between P and B); 3, nasal tract length (segmented line between S and E following the upper jaw dorsally); 4, oral tract length (segmented line between I and E following dorsally the teeth of the lower jaw).

VTL was measured from lateral radiographs obtained from the piglets (Table S1A–D). For each radiograph, three types of measurements were carried out based on several cranial and soft-tissue landmarks (see illustrations and definitions in Fig. 2): the first measurement, skull length, is based on traditional skull morphometry and corresponds to the distance between the prosthion (P) and the basion (B) (Fitch, 2000c). The two other measurements of VTL aim to evaluate the piglets' airway length anterior to the larynx (following the path of sound emitted from the vocal folds). Here, nasal tract length corresponds to the distance between the tip of the snout (S, defined as the projection from the nasal airway onto a line connecting the two apexes of the piglet snout: see Fig. 2C) following the upper jaw dorsally and then the airway down to the base of the epiglottis (E) within the larynx, which marks a clear sharp inflexion point in the airway between the pharynx and the tracheal portion of the airway. Oral tract length corresponds to the distance between the lower incision (I) following dorsally the teeth of the lower jaw and then the airway down to the same E.

In order to account for the uncertainty sometimes caused by low absorbance and scan blurriness (due to slight animal movements during radiograph capture), a quality assessment was made for each radiograph (1: certain, 2: intermediate, 3: unclear), providing a way to easily search for potential outliers and/or errors in the later statistical analysis.

All measurements from radiographs were made using ImageJ (v2.0.0-rc-15-1.49k). DICOM files were loaded in ImageJ, fine-scaled based on DICOM metadata, adjusted for optimal visualization of the landmarks, and measurements were made on segments (PB) or segmented lines (SE and IE). A second measurement session, blind to the first session, was conducted on 10% of the data (based on a random selection excluding the scans labeled with ‘quality 3’ during the first session, as the quality bias is taken into account by the statistical analysis – see below). This resulted in an overall agreement of 99.9% (Pearson's *r*=0.9993), illustrating the reliability of this measurement procedure. The accuracy of the measurements was very high: the mean absolute measurement error ranged from 0.046 to 2.48 mm (mean=0.8 mm) and represented between 0.03% and 1.6% (mean=0.6%) of the overall length, which is negligible compared with the average variation found between individuals of the same age (coefficient of variation ranging from 4.2% to 9.5%) and between series (coefficient of variation ranging from 10.3% to 36.2%).

### Statistical analysis

Prior to analyses, all parameter units were chosen to avoid scaling issues (all frequency parameters are expressed in kHz, length parameters are in cm and weight is in kg). Data normality was assessed using a Shapiro–Wilk test; afterwards, pairwise correlations were computed. Principal component analyses (PCA) were run on groups of variables that were highly correlated and thus redundant with respect to the acoustic and anatomical measurements. Two different PCA were run, one grouping skull, nasal tract and oral tract length into a single VTL component (eigenvalue=2.96, explaining 98.7% of the variance), the other grouping *F*_1_ and *F*_2_ into one ‘formant’ or Fn component (eigenvalue=1.93, explaining 96.8% of the variance). VTL and Fn components were also assessed for normality and then correlations among all variables were computed. Δ*F* was maintained as an individual measurement as it represents a relative measure of *F*_1_ and *F*_2_ variation and could give insight into how evenly/differently formants change through time.

Three types of analysis were conducted, respectively on purely anatomical correlations (testing the effect of BW on VTL), anatomical–acoustical correlations (testing the effect of VTL on formant characteristics) and acoustic allometry (testing the effect of BW on formant characteristics).

To evaluate statistical significance and relative predictive power, data were analyzed by means of model selection using linear mixed models (LMMs) with restricted maximum likelihood estimation (REML) and/or generalized linear mixed models (GLMMs). Models were computed including non-intercorrelated fixed effects and random effect intercepts. Based on visual inspection of the data, models were also run including random slopes for the effect of the main factor of interest (VTL for the anatomical–acoustical dependency, and BW for the anatomical and acoustic allometry dependencies). Our model selection procedure followed a stepwise removal of fixed effects, evaluating a decrease in Akaike information criterion (AIC) scores (corresponding to an improvement of the model), to reach the best model with the lowest AIC. Statistical significance of the final models was evaluated using likelihood ratio tests (final model versus null model, excluding the fixed effect for which significance was being tested; following Winter, 2013). Provided residuals were normally distributed, this model was considered to be validated. Otherwise, a GLMM fitting the dependent data distribution was computed, including the same fixed and random effects/slopes as in the LMM (see Table S2 for details on initial model composition).

To control for the effect of potentially significant errors in the measurements, the same overall analysis was conducted on a reduced sample, excluding the cases in which the quality of one of the three measurements was ranked as low with ‘3’. Data was prepared in SPSS Statistics (v21.0) and statistical analyses were conducted using SPSS and R (http://www.R-project.org/) with the R-package lme4 (Bates et al., 2015). Two-tailed *P*-values are reported with the significance level set at 0.05.

### Ethical note

All procedures were approved by the institutional ethics committee in accordance with GSP guidelines and national legislation (ref. 12/07/97/2014).

## RESULTS

Examination of normality revealed that all variables measured were non-normally distributed. Therefore, non-parametric Spearman rank correlations were computed, which showed that all measured variables were significantly intercorrelated (*P*<0.001 for all correlations; Table 1). Overall, the two components resulting from the PCA have higher correlations with other variables than variables singled out from the components [e.g. Fn correlates better than *F*_1_ and *F*_2_ with VTL and log_10_ of body weight (hereafter, log BW)], justifying the use of the PCA variables. Because we were generally interested in determining the predictability of one variable by another, and because when compared with Fn, Δ*F* showed less strong correlations with both BW and VTL (Table 1), Fn was the only frequency-related variable retained for further analysis (moreover, formant dispersion is usually based on an average of more than three formants, and cannot be appropriately calculated here as only *F*_1_ and *F*_2_ could be clearly distinguished). Finally, log BW was used rather than BW because volume is proportional to the cube of a linear dimension (BW was the only variable log-transformed as the relationships between log BW and VTL and between log BW and Fn appeared to be linear after visual inspection).

**Table 1.**
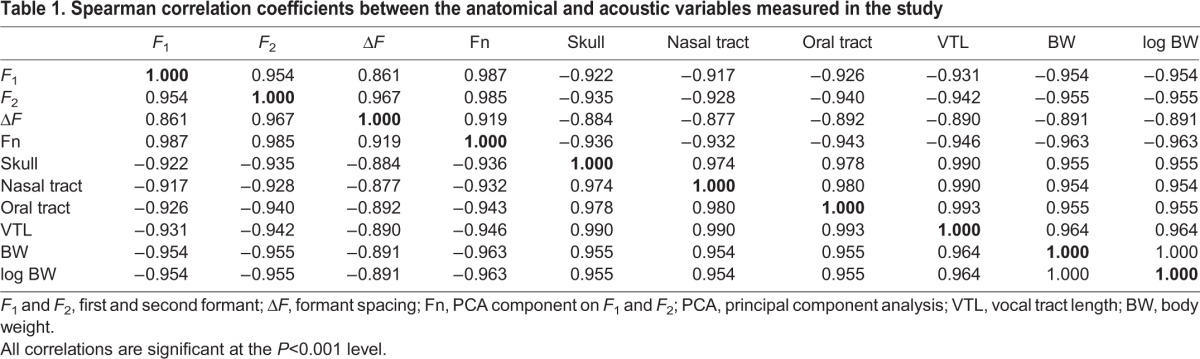
**Spearman correlation coefficients between the anatomical and acoustic variables measured in the study**

### Anatomical dependencies: BW predicts VTL

**Fig. 3.**
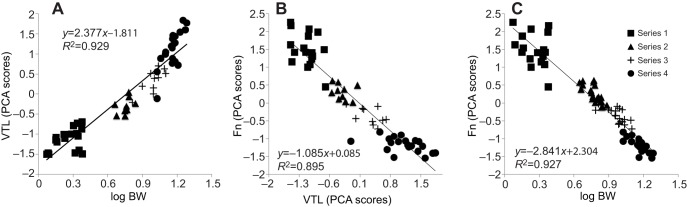
**Bivariate plots illustrating intercorrelations of body weight, VTL and formants.** (A) VTL (PCA scores from a PCA on skull length, nasal tract length and oral tract length) against log body weight (BW, in kg); *N*=60. (B) Formants (Fn; PCA scores from a PCA on *F*_1_ and *F*_2_) against VTL; *N*=57. (C) Fn against log BW (in kg); *N*=77.

Because log BW and VTL were strongly and positively correlated (*r*=0.964, *P*<0.001; Fig. 3A), we further examined the dependence of VTL on log BW with linear models. log BW, Litter (B, R or Z) and Sex (male or female) were entered as fixed effects whereas Individual and Series (1, 2, 3 or 4) were entered as random effects. Two types of model were calculated, either specifying random slopes for the by-Individual and by-Series effect of log BW, or only for the by-Series effect of log BW (based on visual inspection of the data prior to running the analysis; see Table S2 for initial model composition). After stepwise removal of the fixed effects based on a decrease in AIC scores, the best-fitting model was a GLMM (because the residuals from the LMM were non-normally distributed) with a gamma distribution and an inverse link function, including only log BW as fixed effect and random slope only for the by-Series effect of log BW (Table 2). We thus found that BW was the only significant predictor of VTL (*N*=60; predictions not back transformed: β=−1.515, s.e.m.=0.48, *t*=−3.158, *P*=0.002), excluding an effect of sexual dimorphism on this relationship. Inspection of the initial GLMM confirmed the selection of our final model, as neither sex nor litter effects were significant (*P*>0.9). The same analysis was conducted controlling for Age instead of Series and produced the same final model (which is not surprising considering that series number increased in time and was tightly linked to age). Because this study is a longitudinal sampling of the same individuals, our analysis shows that in domestic pigs, the growth of the vocal tract is dependent on BW entirely with no additional significant effects of Sex or Age.

**Table 2.**
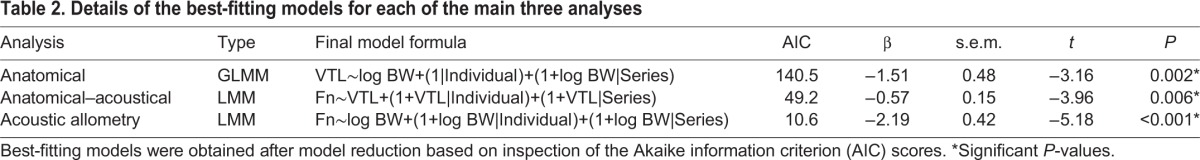
**Details of the best-fitting models for each of the main three analyses**

### Acoustical dependencies: VTL predicts formants

VTL and Fn were strongly negatively correlated (*r*=−0.946, *P*<0.001; Fig. 3B), as predicted based on acoustic principles, and we thus further examined the dependence of Fn on VTL (an anatomical-to-acoustic relationship) in a similar way to the previous analysis (Table S2). Our best-fitting model revealed that VTL is the only significant determinant (*N*=57; β=−0.574, s.e.m.=0.15, *t*=−3.955, *P*=0.006) of Fn (Table 2), including when Age is controlled for instead of Series. Likelihood ratio tests on initial models excluding one main factor at a time (Winter, 2013) confirmed the selection for our final model, as neither sex nor litter effects were significant (respectively, *P*>0.9 and *P*>0.8). This analysis shows that the observed decrease in formant frequencies with body size (Table 1) depends only on the increase in VTL; again, no sex differences were significant.

### Acoustical allometry: BW predicts formants

Finally, looking at acoustic allometric correlations, Fn depended strongly and negatively upon log BW (*r*=−0.963, *P*<0.001; Fig. 3C), as expected based on the previous two correlations. Following the same procedure for model selection (see Table S2 for initial model), the best-fitting model for this analysis only included a significant effect of log BW (*N*=77; β=−2.191, s.e.m.=0.42, *t*=−5.178, *P*<0.001) on Fn (Table 2). This was again confirmed by likelihood ratio tests on initial models, showing non-significant effects of sex (*P*>0.6) and litter (*P*>0.7). As for the two previous analyses, replacing Series by Age yielded the same final model. This result therefore shows that formants are tightly determined by BW, via the intervening variable of VTL, with no additional significant dependence upon age, litter or sex.

These anatomical and anatomical–acoustical analyses were run a second time, removing all cases where VTL measurements from the radiographs included at least one uncertain measurement (quality ‘3’). While AIC scores and significance values differed slightly from the main analyses, all best-fitting models were the same, indicating that measurements potentially involving greater uncertainty did not affect the fundamental relationships found in the analyses reported above.

### Predictive relationships between VTLs and formants

To evaluate the fit between measured formant frequencies and those predicted for a simple uniform tube closed at one end and open at the other, we compared predicted and measured *F*_1_ and *F*_2_ values. From each average individual *F*_1_ (Table S3A–C) and *F*_2_ (Table S4A–C), the predicted VTL was calculated based on the following equations:
(1)
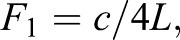

(2)
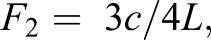


where *c* is the approximate speed of sound in the warm, moist air of a mammalian vocal tract (350 m s^−1^) and *L* is the length of the supralaryngeal tract when considered as a half-open resonant tube (Titze, 1994). Wilcoxon signed-rank tests indicated that the measured nasal tract length and oral tract length were significantly different from the predicted VTL calculated from *F*_1_ (nasal tract length: *Z*=−6.018, *P*<0.001; oral tract length: *Z*=−6.567, *P*<0.001) and *F*_2_ (nasal tract length: *Z*=−6.567, *P*<0.001; oral tract length: *Z*=−6.567, *P*<0.001). Most of our nasal measurements were shorter than predicted from *F*_1_ (*N*=49/57) and all were shorter than predicted from *F*_2_ (*N*=57/57); all of our oral measurements were shorter than predicted from *F*_1_ and *F*_2_. Although highly correlated, measured nasal and oral tract length also significantly differed, and nasal tract length was always longer than oral tract length (*F*_1_: *Z*=−6.567, *P*<0.001; *F*_2_: *Z*=−6.567, *P*<0.001). Thus, although apparently underestimating VTL, our measured nasal tract length was consistently closer to the VTL predicted from *F*_1_ and *F*_2_ than our measured oral tract length [based on the differences between expected values and nasal or oral tract measurements: *F*_1_: *Z*=−6.567, *P*<0.001; *F*_2_ (paired sample *t*-test): *t*_56_=−35.23, *P*<0.001].

Because the vocal folds were not visible in our radiographs, our tracing of nasal and oral tracts stopped at the base of the epiglottis (E), and the full VTL was thus not included. Specifically, the distance between E and the vocal folds (VF, taken at their mid-point) was not included in our measurements, which thus represent a small but consistent underestimate. From digital images of a cross-section of a domestic piglet (W.T.F., unpublished data), we estimated this distance and calculated the resulting increase in VTL. The distance ‘E–VF’ represented, respectively, 8.15% and 9.68% of the nasal and oral tract length stopping at E.

In order to compensate for this additional portion of the vocal tract, we therefore increased our measured nasal and oral tract lengths by 8.15% and 9.68%, respectively (see corrected nasal and oral tract length, Tables S3 and S4) and ran the above analyses again. Nonetheless, as before, the corrected measurements differed from VTL predicted from *F*_1_ (corrected nasal tract length: *Z*=−4.024, *P*<0.001; corrected oral tract length: *Z*=−6.567, *P*<0.001) and *F*_2_ (corrected nasal tract length: *Z*=−6.567, *P*<0.001; corrected oral tract length: *Z*=−6.567, *P*<0.001). Most of the corrected nasal measurements were still shorter than predicted (*F*_1_: *N*=42/57; *F*_2_: *N*=59/57) and all corrected oral measurements were shorter than predicted from *F*_1_ and *F*_2_. Corrected nasal tract length was always longer than corrected oral tract length (*F*_1_: *Z*=−6.567, *P*<0.001; *F*_2_: *Z*=−6.567, *P*<0.001) and thus also closer than corrected oral tract length to the predictions from *F*_1_ (*Z*=−6.567, *P*<0.001) and *F*_2_ (*t*_56_=−32.52, *P*<0.001).

## DISCUSSION

While acoustic cues to adult male quality have been shown to vary over time (see Briefer et al., 2010), the data collected in this study represent, to our knowledge, the first attempt at a longitudinal investigation of acoustic allometry. We found that formants measured in grunt vocalizations provide a reliable cue to body size (assessed by BW) in growing domestic piglets. The very strong correlations between VTL, formants and body size (Table 1, Fig. 3), together with the predictive models that we have computed (Table 2), leave little doubt that formants contain accurate information regarding body size, because increasing BW strongly predicts increasing VTL, which in turn predicts decreasing formant frequencies. Crucially, by resampling the same individuals on four occasions and controlling for age and sex, we could disentangle the specific roles of these parameters in pig vocal allometry. We found that formant frequencies were predicted by body size rather than age, and found no suggestions of potential acoustic sexual dimorphism, or vocal tract modification specifically dependent on age in this species and stage of development. Grunt formants could therefore provide relevant information to listeners, provided that these acoustic cues to body size are perceived and used by conspecifics.

### On the origin of formant frequencies within grunts

Estimations of VTL based on *F*_1_ and *F*_2_ (Tables S3 and S4) were invariably closer to the measured nasal tract length than to the measured oral tract length. Measured nasal and oral tract length were always shorter than predicted by *F*_2_ (Table S4A–C). Regarding the VTL predicted by *F*_1_, measured nasal tract length was shorter than predicted in most individuals (*N*=49/57), while measured oral tract length was always shorter than predicted (Table S3A–C). We therefore suggest that grunts for our sample were mostly produced nasally, in accordance with previous cineradiographic observations of grunts by a vocalizing piglet (Fitch, 2000b).

The fact that predicted VTLs do not perfectly match nasal tract measurements can be explained by several factors. First, our calculations and predictions for expected VTL were based on a quarter-wave resonance tube model, which assumes a closed end (at the glottis) and an open end (the mouth for the oral tract, the nostrils for the nasal tract; Titze, 1994). This does not take into account the changing cross-sectional area (or ‘shape’) of the vocal tract, which is also important in determining formant frequencies and could partly explain the difference between observed and expected VTLs. However, we expect the effect of vocal tract shape to be negligible based on these and previous X-ray observations (Fitch, 2000a,b); furthermore, the effect, if present, would equally concern the nasal and oral airways and thus does not modify our analysis and conclusion. Second, VTL measurements were made down to the base of the epiglottis, which was clearly visible in our radiographs. However, according to the source-filter theory of voice production (Fant, 1960), sound is produced by the vibrating vocal folds (whose vibration rate defines *F*_0_) and then filtered by the supralaryngeal tract (enhancing formants). When correcting our initial measurements for the missing distance between the base of the epiglottis and the vocal folds, we reached similar conclusions, with measurements still typically shorter than predicted. Another potential reason is that laryngeal position in domestic pigs is not as static as previously thought (Fitch, 2000b) and larynx position could thus descend during vocalization (and thus contain lower formants) when piglets produce grunts compared with when they remain silent (which was typically the case during radiographs). Finally, in a few cases nasal tract length was longer than predicted by *F*_1_: this could also be explained by laryngeal mobility and our experimental setup. Although we tried to keep piglets as calm as possible while proceeding with radiographs, in some cases piglets produced squeals while being scanned. Squeals in the domestic pig are very loud calls, which involve retracting the larynx down from the nasopharyngeal region (Fitch, 2000b), in turn leading to a fully extended supralaryngeal tract. Measurements of radiographs of the VTL in this configuration would therefore exceed that characteristic of a grunt call and could explain these isolated observations.

It should be noted that in this study we investigated how formants, instead of formant dispersion, predict body size. These measures are of course intimately related, and it has been suggested that while individual formants could provide information regarding VTL, they are more liable to uncontrolled variability due either to movements or to deviations from the uniform tube assumption (Fitch, 1997; Owren et al., 1997); formant dispersion, in contrast, relies on the redundancy of formant spacing pattern and is thus expected to be more robust (Fitch, 1997). As a result, rather than focusing on individual formant measurements (Owren et al., 1997), most studies investigating formant-related characteristics in mammal vocal communication have used some variant of formant dispersion (Charlton et al., 2011b, 2009a; Fischer et al., 2004; Fitch, 1997; Reby and McComb, 2003; Sanvito et al., 2007). In the present study, information redundancy was low because we were only able to measure the first two formants consistently. Furthermore, the grunts extracted from our labeling were chosen to be as stable and consistent as possible, minimizing the problem raised by formant variability through time. Finally, because grunts appeared to be produced nasally, acoustic attenuation could have occurred as a result of higher sound absorbance from nasal cavities (Fitch, 2000b) or the generation of antiformants by the closed mouth cavity (Kurowski and Blumstein, 1987), explaining why only two formants were clearly distinguishable.

### Selection pressures and grunt-specific cues

Previous work has shown that two main call types, grunts and squeals, could be consistently identified while investigating the vocal repertoires of both domestic pigs (Kiley, 1972; Tallet et al., 2013) and wild boars (Garcia et al., 2016; Klingholz et al., 1979). Unlike squeals, the acoustic characteristics of grunts make them particularly well suited for highlighting formants because of their low *F*_0_ (Fitch and Hauser, 1995; Ryalls and Lieberman, 1982), even though the nasal production typical of this call type might slightly impair our ability to track formants compared with formants from calls of other mammalian species (Charlton et al., 2011b; Reby and McComb, 2003).

Grunts are produced across various contexts in which extracting information about the caller might prove beneficial to the receiver. Grunts are, for instance, produced by male domestic (Kiley, 1972) and wild (Meynhardt, 1990) boars as a courtship display, and as an alarm signal in female wild boars (Klingholz and Meynhardt, 1979; Klingholz et al., 1979). It has been shown in various taxa that body size often plays a major role in sexual selection (Carranza, 1996; Clutton-Brock, 2009; Clutton-Brock et al., 2006; Hedrick and Temeles, 1989; Ryan, 1985), and body size influences resource holding potential and fighting ability in mammals on both a within-species and a between-species level (Clutton-Brock et al., 1979; Morton, 1977; Persson, 1985), including in domestic pigs (Jensen, 2002). Advertising body size in such contexts may be beneficial for large individuals, and the results of the current study suggest that, presumably originating in wild boar vocalizations, the domestic pig grunt can provide a cue to the signaler's body size. Furthermore, retrieval of this information should be biologically relevant to conspecifics (both in sexual competition and in agonistic group encounters, as documented by Meynhardt, 1990), which suggests that pigs should both perceive and attend to formants in conspecific vocalizations. Playback experiments, preferably using resynthesized grunts in which the formants are shifted to simulate different phenotypes, would be necessary to test this prediction.

In several mammalian species, the selective pressures on body size advertisement appear to have led to specific vocal tract adaptations that allow exaggeration of the acoustic impression of body size via formant lowering. Some examples include laryngeal retraction down to the sternum (Fitch and Reby, 2001) or possibly even into the thoracic chamber (Charlton et al., 2011b), the presence and inflation of vocal air sacs (Harris et al., 2006) and rostral extension of a nasal vestibulum (Frey et al., 2007). Our results combined with previous radiographic observations strongly suggest that domestic pig grunts are produced nasally. Because measured nasal tract length was consistently longer than measured oral tract length, this implies that lower formants would be produced from nasal grunts than expected from grunts produced orally, potentially indicating a mild form of body size exaggeration.

We note a previous speculation that the sound source in at least some grunts could be a dorsal velar closure (‘snoring’) rather than vocal fold vibrations (Klingholz et al., 1979). We know of no data relevant to this speculation. Whether such a non-standard production mechanism would have an effect on formants in the context of size exaggeration would require further in-depth investigation of the production mechanisms of this vocalization.

In addition to the agonistic or courtship contexts mentioned above, grunts are also used more generally as contact calls, noticeably occurring during foraging and nursing events in domestic pigs (Kiley, 1972) and wild boars (Klingholz et al., 1979). In both of these contexts, individuality appears to be another type of potentially useful acoustic information. It has indeed been shown in several species that contact calls contain cues to individual identity (Favaro et al., 2015; Jansen et al., 2012; Müller and Manser, 2008; Shapiro, 2009; Townsend et al., 2010). In meerkats and banded mongooses for instance, individual-specific information is used by conspecifics during foraging for vigilance and coordination purposes (for a review, see Manser et al., 2014). Given the strong similarities with the social and vocal communication system found in pigs and meerkats and banded mongooses (also highly social and vocal mammals; Manser et al., 2014), it is reasonable to suggest that cues to individual identity might be perceived and used by other conspecifics in domestic pigs. Parent–offspring recognition is another situation typical of the socio-communicative system characterizing this species where cues to individuality could exist, as such recognition relies on vocal communication in other mammalian species (Briefer and McElligott, 2011; Charrier et al., 2001; Fischer, 2004; Insley, 2001). Previous work on domestic pigs indeed reported that grunts produced during nursing allowed litter discrimination by sows (Illmann et al., 2002) and suggested mother recognition by piglets based on formant-related acoustic features (Schön et al., 1999). Together with our results, this suggests that grunt formants have the potential for carrying multiple messages, as seen in other mammals [rhesus macaques (Fitch, 1997; Rendall, 1996), koalas (Charlton et al., 2012, 2011a)]. Again, playback studies would be required to test this hypothesis.

In conclusion, our results show that formants in domestic piglet grunts are a reliable indicator of body size throughout piglet development. These acoustic cues are available and would in theory be useful to the receiver in various contexts such as sexual selection and agonistic interactions. However, whether information related to vocal tract filtering is perceived and used by conspecifics, including in the case of multi-message signaling, remains unknown. Future research involving playback experiments combined with formant manipulation and signal re-synthesis should improve our understanding of the mechanisms involved in perception and interpretation of domestic pig grunts by their conspecifics. This would in turn provide additional insight regarding the selective pressures, such as sexual selection and/or size exaggeration, acting upon this species' communication system. Because domestic pigs are common, highly vocal and easy to work with, they provide excellent potential as a study species for future bioacoustics research, especially given that their wild progenitors, wild boars, still exist and remain both widespread and relatively accessible.
